# Genome-wide identification and expression analysis reveals spinach brassinosteroid-signaling kinase (BSK) gene family functions in temperature stress response

**DOI:** 10.1186/s12864-022-08684-5

**Published:** 2022-06-20

**Authors:** Yang Li, Heng Zhang, Yongxue Zhang, Yanshuang Liu, Yueyue Li, Haodong Tian, Siyi Guo, Meihong Sun, Zhi Qin, Shaojun Dai

**Affiliations:** 1 Development Center of Plant Germplasm Resources, College of Life Sciences, Shanghai Normal University, Shanghai, 200234 China; 2 Key Laboratory of Saline-alkali Vegetation Ecology Restoration (Northeast Forestry University), Ministry of Education, College of Life Sciences, Northeast Forestry University, Harbin, 150040 China; 3 State Key Laboratory of Crop Stress Adaptation and Improvement, School of Life Sciences, Henan University, Kaifeng, 475004 China

**Keywords:** Spinach, BSK family, Expression pattern, BR signaling, Heat stress

## Abstract

**Background:**

Brassinosteroid (BR)- signaling kinase (BSK) is a critical family of receptor-like cytoplasmic kinase for BR signal transduction, which plays important roles in plant development, immunity, and abiotic stress responses. Spinach (*Spinacia oleracea*) is cold- tolerant but heat- sensitive green leafy vegetable. A study on BSK family members and BSKs- mediated metabolic processes in spinach has not been performed.

**Results:**

We identified and cloned seven *SoBSKs* in spinach. Phylogenetic and collinearity analyses suggested that SoBSKs had close relationship with dicotyledonous sugar beet (*Beta vulgaris*) rather than monocotyledons. The analyses of gene structure and conserved protein domain/ motif indicated that most SoBSKs were relative conserved, while SoBSK6 could be a truncated member. The prediction of post-translation modification (PTM) sites in SoBSKs implied their possible roles in signal transduction, redox regulation, and protein turnover of SoBSKs, especially the N-terminal myristoylation site was critical for BSK localization to cell periphery. *Cis*-acting elements for their responses to light, drought, temperature (heat and cold), and hormone distributed widely in the promoters of *SoBSKs*, implying the pivotal roles of *SoBSKs* in response to diverse abiotic stresses and phytohormone stimuli. Most *SoBSKs* were highly expressed in leaves, except for *SoBSK7* in roots. Many *SoBSKs* were differentially regulated in spinach heat- sensitive variety Sp73 and heat- tolerant variety Sp75 under the treatments of heat, cold, as well as exogenous brassinolide (BL) and abscisic acid (ABA). The *bsk134678* mutant Arabidopsis seedlings exhibited more heat tolerance than wild- type and *SoBSK1*- overexpressed seedlings.

**Conclusions:**

A comprehensive genome- wide analysis of the BSK gene family in spinach presented a global identification and functional prediction of *SoBSKs*. Seven SoBSKs had relatively- conserved gene structure and protein function domains. Except for SoBSK6, all the other SoBSKs had similar motifs and conserved PTM sites. Most SoBSKs participated in the responses to heat, cold, BR, and ABA. These findings paved the way for further functional analysis on BSK- mediated regulatory mechanisms in spinach development and stress response.

**Supplementary Information:**

The online version contains supplementary material available at 10.1186/s12864-022-08684-5.

## Introduction

Spinach (*Spinacia oleracea*) is an important and nutritious green leafy vegetable, which belongs to the Amaranthaceae family [1]. Generally, spinach is cold- tolerant but heat- sensitive vegetable species, which limits its distribution and production in tropics [2]. Under 37 °C/ 32 °C (day/night) treatment for 24 h, we found that the relative water content in leaves from heat- sensitive variety Sp73 was decreased to 66.1%, which was obviously lower than that in heat- tolerant variety Sp75 (75.7%) [3, 4]. The genomics analysis on spinach presented a high-quality chromosome- scale reference genome, and the transcriptome sequencing and population genomic investigations discovered the genome architecture, evolution, and domestication, which was valuable for understanding the genetic basis of important agronomic traits and breeding [1, 5]. Besides, high- throughput proteomics analysis on spinach varieties of Sp73 and Sp75 revealed that heat perception, signal transduction, and reactive oxygen species (ROS) scavenging were crucial for the heat tolerance [3, 4]. However, the finetuned heat- responsive molecular mechanism in spinach keeps unknown due to the lack of efficient genetic transformation system [6].

Brassinosteroids (BRs), a group of steroid hormones, regulate diverse physiological processes upon plant development and stress response [7]. BR signal transduction in Arabidopsis has been well addressed [8]. BR signal is precepted by leucine-rich repeat receptor- like kinase BR INSENSITIVE1 (BRI1) and coreceptor BRI1- ASSOCIATED RECEPTOR KINASE1 (BAK1) at the cell surface [9, 10]. The activated BRI1 by sequential transphosphorylation from BAK1 can phosphorylate BR SIGNALING KINASE (BSK) and subsequently enhance BRI1 SUPPRESSOR 1 (BSU1) activity [11, 12], and then the activated BSU1 inhibits BRASSINOSTEROID INSENSITIVE2 (BIN2) through dephosphorylation BIN2, promoting the unphosphorylated BRASSINAZOLE RESISTANT1/bri1 EMS SUPPRESSOR1 (BZR1/BES1) transcription factors to accumulate and enter the nucleus for regulating BR- targeted gene expression [13, 14].

BSK is a critical family of receptor-like cytoplasmic kinase (RLCK) in the initial step, activating downstream phosphatase BSU1 for BR signal transduction [12]. Three Arabidopsis BSKs (i.e. AtBSK1, AtBSK2, and AtBSK3) have been identified as BR- responsive proteins [11]. The functions of some members among 12 Arabidopsis AtBSKs and five rice OsBSKs have been well investigated, and they all have conserved kinase catalytic domain at N-terminus and tetratricopeptide repeats (TPRs) domain at C-terminus [11, 15–17]. Besides, myristoylation site at the N-terminus for plasma membrane (PM)- localization, critical serine (S) and lysine (K) sites (e.g., S^230^ and K^104^ in AtBSK1), as well as sumoylation and ubiquitination sites were also conserved in most AtBSKs [17].

BSKs were involved in BR signal transduction but also other diverse signaling pathways [18, 19]. Upon plant growth and development, several BSKs (i.e. BSK3, BSK4, BSK6, BSK7, and BSK8) were partially redundant in BR signaling [20]. However, BSK3 was the only reported member for BR- mediated plant root growth [21] and foraging under low nitrogen [22]. On the contrary, BSK12 (also called SHORT SUSPENSOR, SSP) activated the YODA mitogen-activated protein kinase (MAPK) pathway, but not BR signaling, upon embryogenesis of Arabidopsis [23]. Under salinity stress, overexpression of *BSK5* inhibited the expression of abscisic acid (ABA) deficient3 (*ABA3*) and 9-*cis*-expoxycarotenoid dioxygenase (*NCED3*) for ABA biosynthesis in Arabidopsis [24]. Besides, BSK5 promoted BES1-PHYTOCHROME INTERACTING FACTOR4 (PIF4)/PIF5 module- mediated auxin signaling for plant hypocotyl elongation under shade (low ratio of red to far-red light), but this enhancement was blocked by NaCl- induced ABA signaling [25].

During plant immunity response, BSK1 positively regulated pathogen- associated molecular pattern (PAMP)- trigged immunity (PTI) (i.e. *edr*- mediated resistance) by physically associating with the PAMP receptor FLAGELLIN SENSING2 (FLS2) in response to FLS2- mediated ROS bursting [26, 27]. BSK1- FLS2 module did not activate MAPK signaling, but BSK1 directly phosphorylated S^289^ in MAPK kinase kinase 5 (MAPKKK5) to form immune complex for triggering the MAPK cascade [28]. During the resistance to bacterial pathogens, the immune signals can be transmitted from RECEPTOR-LIKE KINASE 902 (RLK902) to BSK1 through the phosphorylation of BSK1 at S^230^ [29], which was also the key phosphorylation site for BR signaling [11]. Besides, BSK5 can interact with 11 immunity- associate receptor- like kinases (e.g., STRUBBELIG-RECEPTOR FAMILY (SRF)6/ 7, FERONIA, WALL-ASSOCIATED RECEPTOR KINASE-LIKE (WAKL)8/ 14/ 18, BAK1-INTERACTING RECEPTOR-LIKE KINASE1 (BIR1), LYSIN-MOTIF RECEPTOR KINASE5 (LYK5), PEP1 RECEPTOR1 (PEPR1), ERECTA, and SUPPRESSOR OF BIR1–1 (SOBIR1)) for promotion of multiple PAMPs/ damage- associated molecular patterns (DAMPs)- induced PTI processes [18, 30]. In addition, BSK8 was associated with FLS2 [31] and phosphorylated on flg22 treatment [32], although its molecular mechanism was still unclear. On the other hand, in the process of effector- triggered immunity (ETI) initiation, PM- localized BSK1 and BSK8 were identified as the components of Ribosomal protein S2 (RPS2) protein complex [31]. RPS2 is a well- studied resistance protein for the perception of pathogen. Therefore, BSKs played key roles in pathogen perception.

Interestingly, the dynamic spatiotemporal reorganization of BSK1 within PM was a crucial node for signal- specific activation of the balance between growth and immunity [33]. BSK1 multimerization and dissociation from the complexes of FLS2-BSK1 or BRI1-BSK1 were induced by the flg22 or exogenous BR treatment, respectively. Moreover, the flg22- triggered BSK1 translocated from membrane rafts to non- membrane raft regions for immunity response, whereas BR- induced BSK1 remained in membrane rafts for growth regulation [33].

Besides of Arabidopsis, several BSKs from other plants were reported to function in diverse biological processes, such as the sprouting of potato (*Solanum tuberosum*) [34], drought responses of maize (*Zea mays*) [35], wild barley (*Hordeum spontaneum*) [36] and Kentucky bluegrass (*Poa pratensis*) [37], cold tolerance of *Populus tomentosa* [38], as well as immune response of rice (*Oryza sativa*) [15] and tomato (*Solanum lycopersicum*) [39]. However, the BSK- regulated heat- responsive mechanisms in spinach were still largely unclear.

In this study, seven members of the spinach *BSK* gene family were identified by screening the spinach whole- genome database [1, 5]. The phylogenetic trees were constructed along with their homologous genes from sugar beet (*Beta vulgaris*), Arabidopsis, rice, maize, as well as common tobacco (*Nicotiana tabacum*) and woodland tobacco (*Nicotiana sylvestris*) for evaluating the evolution relationship and classification. The chromosomal distribution, gene structure, conserved function domains/ sites, and subcellular localization of SoBSKs were analyzed. Moreover, we examined the expression patterns of *SoBSK*s in different organs from heat- tolerant variety Sp75 and heat- sensitive variety Sp73, and upon exposure to temperature stresses and exogenous hormone treatments. Furthermore, diverse PTMs were found in several SoBSKs, implying the potential functions of SoBSKs. All these results lay a solid foundation for understanding the SoBSK- mediated molecular mechanisms in spinach development and stress response.

## Results

### Phylogenetic relationship of BSKs from spinach and other plant species

**Fig. 1:**
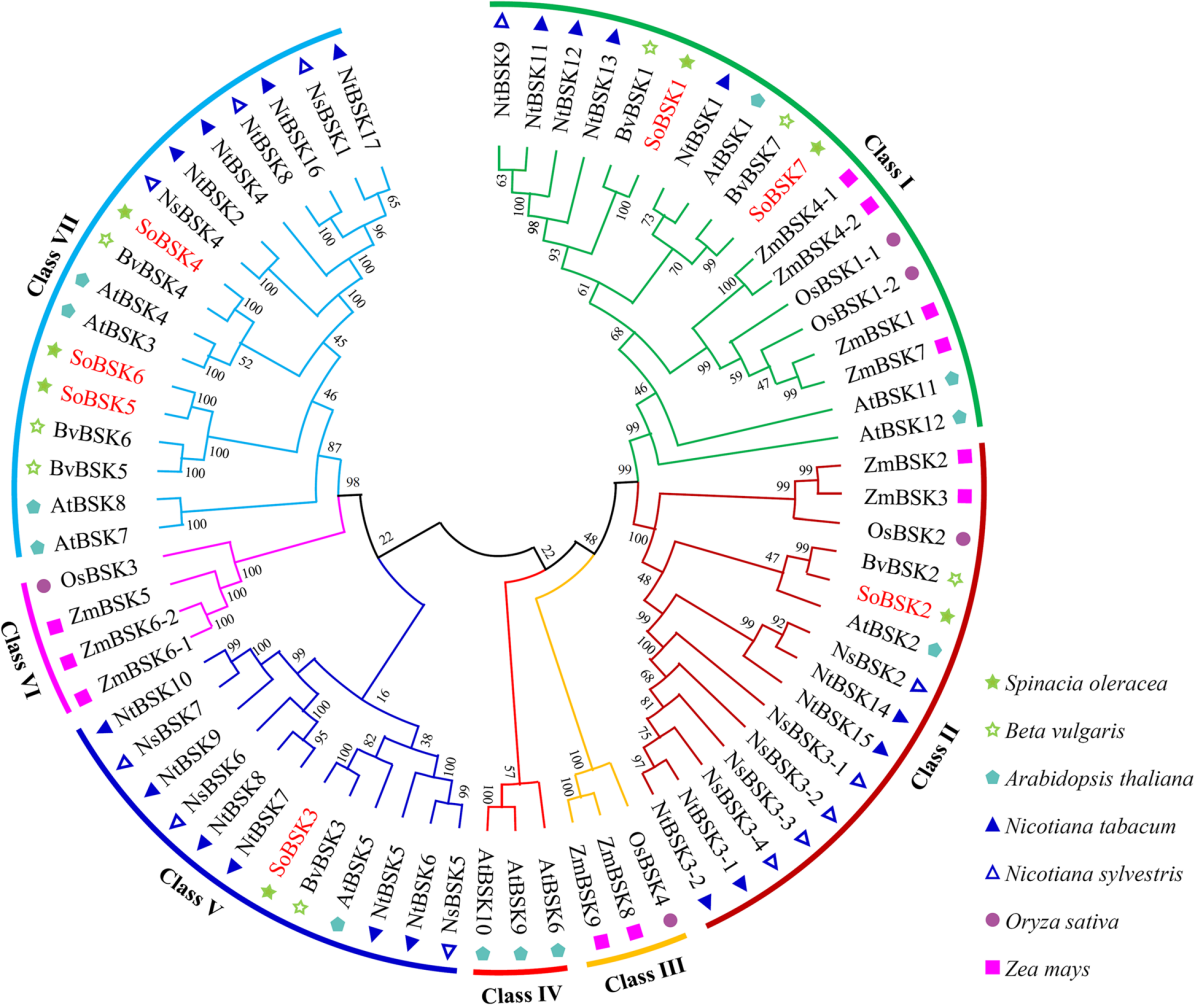
Phylogenetic analysis of brassinosteroid-signaling kinases (BSKs) from spinach and other six representative plants. The phylogenetic tree was built based on the complete amino acid sequences of the BSKs by MEGA4-X with the maximum likelihood method, which indicated the phylogenetic relationships among 72 BSKs from spinach (*Spinacia oleracea*), sugar beet (*Beta vulgaris*), *Arabidopsis thaliana*, rice (*Oryza sativa*), maize (*Zea mays*), as well as common tobacco (*Nicotiana tabacum*) and woodland tobacco (*Nicotiana sylvestris*)*.* The colored ranges indicated the total BSK proteins classed into seven classes. Various shapes represented different plant species. The accession numbers, coding sequences (CDSs), and amino acid sequences of these BSKs were listed in Additional file 1. Bootstrap = 1000

To evaluate the evolution relationship of spinach BSKs with others, seven spinach BSKs, together with 65 BSKs from six representative model plants and/ or crops, were used to construct an un-rooted phylogenetic tree (Fig. 1). The protein sequences of seven BSKs from spinach, seven from sugar beet, 12 from Arabidopsis, 18 from common tobacco, 12 from woodland tobacco, five from rice, and 11 from maize were clustered in the phylogenetic tree (Fig. 1 and Additional file 1). Among them, 12 Arabidopsis AtBSKs [11, 17–32, 40, 41], five rice OsBSKs [15, 16], two woodland tobacco NsBSKs (i.e. NsBSK1 and NsBSK3) [42], a common tobacco NtBSK2 [42], and nine maize ZmBSKs [35] have been reported previously, and all the other sequences of BSKs were obtained by searching against the genome database using BLASTp.

Totally, 72 BSKs were grouped into seven classes (Class I to Class VII) (Fig. 1). Among them, seven SoBSKs distributed into four classes (Class I, Class II, Class V, and Class VII), while seven BSKs from monocotyledon rice and maize (in Class III and Class VI), as well as three BSKs from dicotyledon Arabidopsis (in Class IV) were grouped into the rest three classes, respectively (Fig. 1). Seven SoBSKs were grouped into unique subgroups with their homologous BSKs from sugar beets and Arabidopsis, but not tobacco species, while most BSKs from two tobacco species clustered together, except for NtBSK1 from common tobacco classed with AtBSK1 from Arabidopsis in a subgroup of Class I. The rest nine BSKs from monocotyledonous rice and maize distributed into two individual subgroups in Class I and Class II. Thus, the phylogenetic analysis indicated that these BSKs were highly conserved among monocotyledons and dicotyledons. Moreover, seven SoBSKs were closely homologous with BvBSKs from sugar beet, and also close to Arabidopsis BSKs rather than those from two tobacco species. This is consistent with the comparative genomics analysis and their evolutionary relationships [1].

### Chromosomal distribution analysis of SoBSKs

**Fig. 2:**
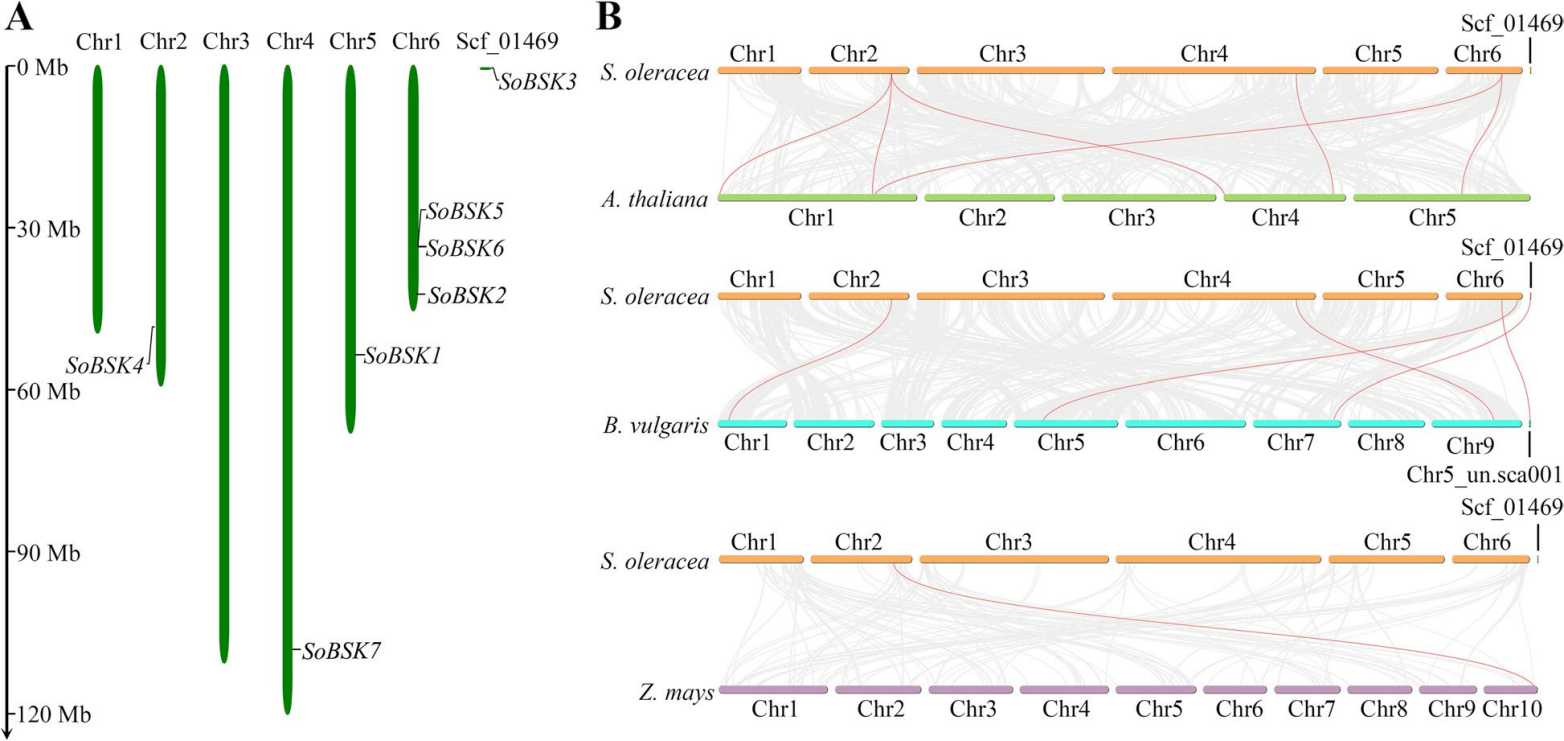
Distribution of *SoBSKs* on chromosomes and collinearity analysis of *BSKs* between spinach and three representative plant species. **A** Distribution of *SoBSKs* on chromosomes. The diagram was drawn using the MapGene2Chrom web v2 software. Six *SoBSK* genes were located on four chromosomes and *SoBSK3* was mapped onto an unanchored scaffold (Scf_01469). The vertical bars marked the chromosomes of spinach. The scale on the left represented the chromosome length. **B** Collinearity analysis of *BSKs* among spinach, Arabidopsis, sugar beet, and maize. Gray lines in the background indicated the collinear blocks within spinach and other plant genomes, while the red lines highlighted the syntenic *BSK* gene pairs

Chromosomal distribution analysis suggested that seven *SoBSKs* were distributed nonrandomly and unevenly across four out of six spinach chromosomes, except for chromosome 1 and 3 (Fig. 2A). *SoBSK4*, *SoBSK7*, and *SoBSK1* were localized on chromosome 2, 4, and 5, respectively, while chromosome 6 harbored *SoBSK2*, *SoBSK5*, and *SoBSK6*. In addition, *SoBSK3* was mapped onto an unanchored scaffold (Scf_01469) according to the draft spinach Sp75 genome, which was assembled with low coverage rate (47%) [1].

### Collinearity analysis of BSKs between spinach and other plants

The collinearity relationship was constructed between spinach and three plant species (i.e. Arabidopsis, sugar beet, and maize) (Fig. 2B). Collinearity analysis showed that *SoBSKs* had homologous genes in Arabidopsis, sugar beet, and maize, of which Arabidopsis had six homologous gene pairs located in chromosome 1, 4, and 5, followed by sugar beet (five gene pairs in chromosome 1, 7, and 8, as well as an unanchored scaffold chr5_nu.sca001), and maize (only one gene pair in chromosome 10) (Fig. 2B and Additional file 2). Interestingly, *SoBSK4* (LOC110789384) had homologous gene pairs with all three plant species, especially three gene pairs in Arabidopsis, including *AtBSK3* (AT4G00710), *AtBSK4* (AT1G01740), and *AtBSK7* (AT1G63500). *SoBSK6* (LOC110778653) and *SoBSK7* (LOC110788203) had homologous gene pairs with both sugar beet and Arabidopsis. In addition, both *SoBSK4* and *SoBSK6* had close relationship with *AtBSK7*. *SoBSK6* was also homologous with *AtBSK8* (AT5G41260). These results indicated that *SoBSKs* had close phylogenetic relationship with dicotyledons rather than monocotyledons.

### Gene structure and protein function domain analysis of SoBSKs

**Fig. 3:**
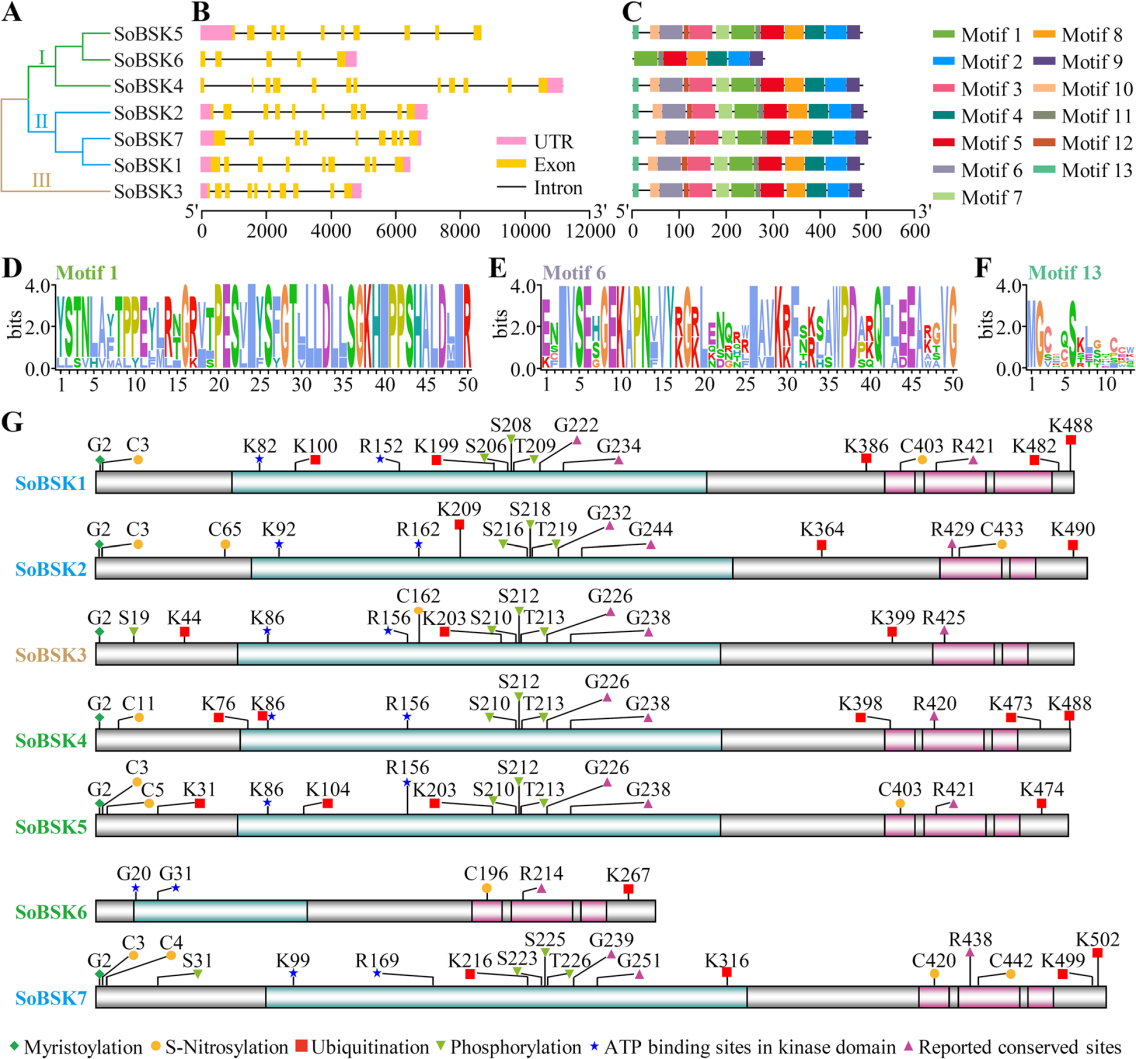
Phylogenetic tree, gene structure, conserved motifs, protein function domains, and conserved modification sites of SoBSKs. **A** Phylogenetic tree of SoBSKs constructed by the Neighbor-Joining method. **B** The exon, intron, and untranslated region (UTR) organization of *SoBSKs*. Light pink boxes indicated UTR, yellow boxes represented exons, and lines showed introns. **C** Conserved motifs of SoBSKs. Various colors represented different motifs. **D-F** The logos showing the conserved residues in motif 1 (**D**), motif 6 (**E**), and motif 13 (**F**). **G** Function domains and conserved modification sites of SoBSKs. Kinase domain and tetratricopeptide repeat (TPR) were labeled in green and pink, respectively. Conserved modification sites were labeled with different shapes. C: Cysteine; G: Glycine; K: Lysine; R: Arginine; S: Serine; T: Threonine

Phylogenetic analysis indicated that SoBSKs were grouped into three groups according to their amino acid sequences. SoBSK5, SoBSK6, and SoBSK4 were included in Group I, SoBSK1, SoBSK7, and SoBSK2 were classed into Group II, while SoBSK3 was a distinct Group III (Fig. 3A).

To predict the evolutionary feature and functional diversification, exon-intron organization of seven *SoBSKs* was analyzed. *SoBSK4* contained eleven introns, *SoBSK2*, *SoBSK3*, and *SoBSK5* had nine introns, *SoBSK1* and *SoBSK7* had eight introns, while *SoBSK6* had four introns (Fig. 3B). The diversity of various introns and exons implied the possible alternative splicing in *SoBSKs* was critical for spinach development and stress response, which were reported in Arabidopsis *BSKs* [17]. In addition, *SoBSK4* and *SoBSK6* only had 3’UTR, *SoBSK5* only had 5’UTR, while the remaining four *SoBSKs* had both 5’UTR and 3’UTR (Fig. 3B).

The conserved motifs analysis suggested that thirteen conserved motifs were found in the six SoBSKs, except for SoBSK6 which only had seven motifs (Motif 1, 2, 4, 5, 8, 9, and 11) (Fig. 3C and Additional file 3). Among 13 motifs, seven motifs (Motif 1, 3, 5, 6, 10, 11, and 12) were localized in the kinase domain, while three motifs (Motif 2, 4, and 9) were distributed in the TPR domain. Importantly, more than half amino acid sites (26 out of 50) in Motif 1 were highly conserved, among of which contained four conserved S sites (S22, S26, S36, and S43) (Fig. 3D). Besides, the 2nd serine in Motif 1 was also conserved in SoBSKs, except for SoBSK6 (Fig. 3D and G). The Arabidopsis homologous site of the 2nd serine in Motif 1 (S^230^ in AtBSK1 and S^210^ in AtBSK3) had been reported to a phosphorylated site by AtBRI1 in the BR signaling pathway [11, 16]. Moreover, the glycine (G)16, G28, and G37 in Motif 1 were also conserved in all SoBSKs (Fig. 3D and G). The homologous sites in AtBSK3 of G16 and G28 (G^226^ and G^238^ in AtBSK3) can interfere other BSKs in BR signaling and protein stability of AtBSK3, respectively [21]. Motif 6 had 26 conserved amino acids out of 50 amino acids, including two lysines (K10 and K29 in Motif 6), and K29 was the key site for ATP binding and BSK kinase activity (Fig. 3E and G) [21]. In addition, the 2nd glycine in Motif 13 was a highly conserved myristoylation site for membrane localization of BSKs (Fig. 3F and G) [16, 18].

### Analyses of conserved function domains and modification sites in SoBSKs

A common feature of BSKs from Arabidopsis and rice is the presence of a N-terminal kinase domain and two or three C-terminal TPR domains, which can interact directly with each other for autoregulating its kinase activity [11, 16]. In spinach, we also found the conserved N-terminal kinase domain and C-terminal TPR domains in seven SoBSKs (Fig. 3G). SoBSK2 and SoBSK3 contained two C-terminal TPR domains, but all the other SoBSKs had three C-terminal TPR domains (Fig. 3G).

We performed the prediction of some conserved amino acid sites for protein PTM. The sequence alignment with these reported functional sites in Arabidopsis [11, 18, 21, 26, 27, 29, 41] and rice [16], as well as the online tools GPS-SNO 1.0 [43] and UbiComb [44], were used for the predictions of phosphorylation, myristoylation, S-nitrosylation, and ubiquitination, respectively (Fig. 3G). Except for SoBSK6, all the other SoBSKs had a serine site and a lysine site in the kinase domain (Fig. 3G), which were the homologous sites with the conserved phosphorylated site by BRI1 and ATP- binding site for kinase activity determination in Arabidopsis, respectively [11, 26]. Similarly, most SoBSKs, except for SoBSK6, had a glycine site for myristoylation modulating its PM localization, and more than one cystine for S-nitrosylation in response to nitric oxide signal [43] (Fig. 3G). In addition, SoBSK1 and SoBSK5 had a predicted ubiquitination site (K^100^ in SoBSK1 and K^104^ in SoBSK5) in the kinase domain (Fig. 3G), which were supposed to be a critical site for BSK degradation through ubiquitin-proteasome system. This indicated that these conserved PTM sites determined subcellular localization, redox regulation, and protein turnover of SoBSKs.

### Analysis of *cis*-acting elements in *SoBSK* promoters

**Fig. 4:**
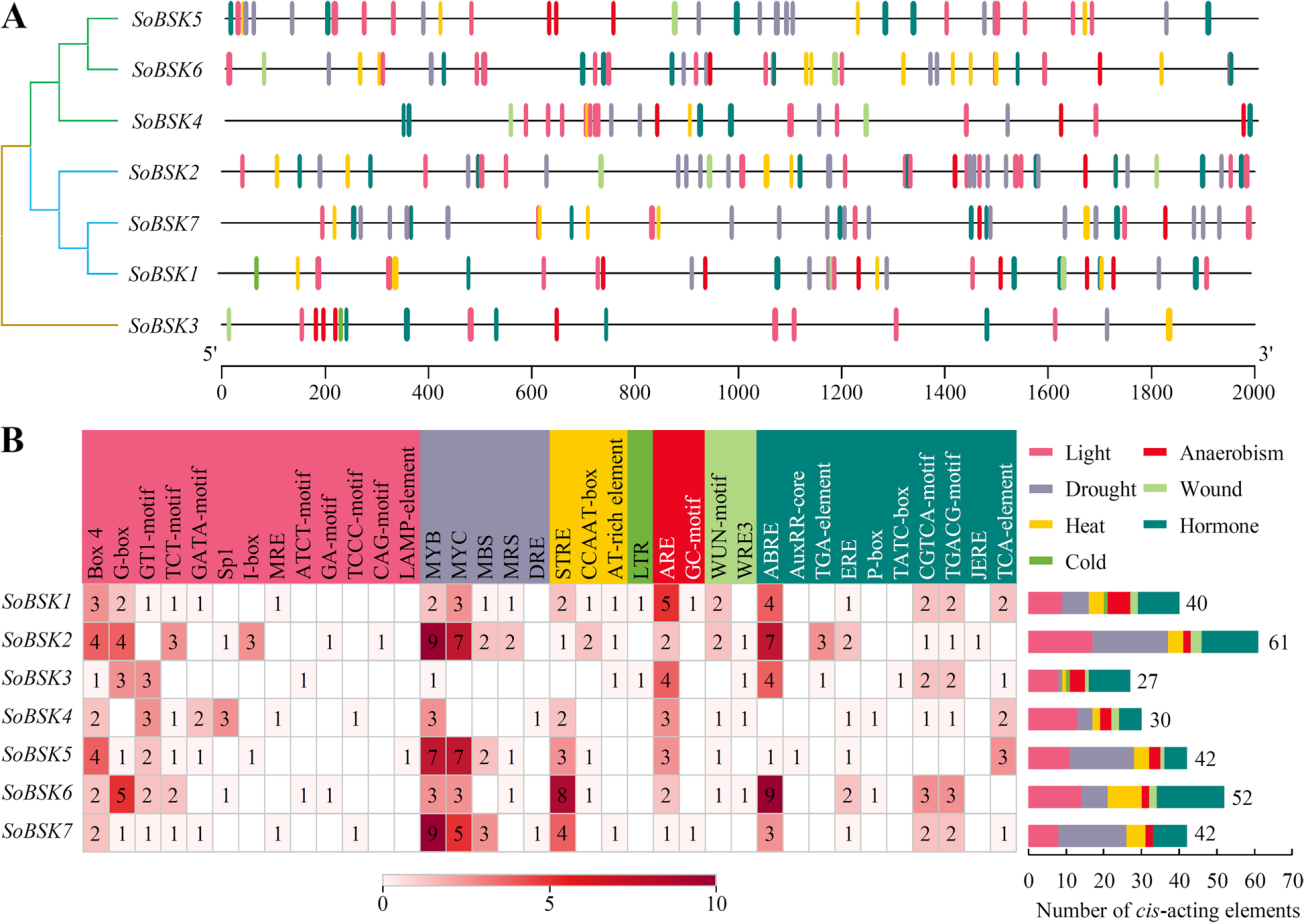
Analysis of the *cis*-acting elements in the promoter regions of *SoBSK* genes. **A** The *cis*-acting elements distribution in *SoBSK* promoters. **B** The names and numbers of *cis*-acting elements in *SoBSK* promoters. The heatmap in grid and the color columns indicated the numbers of *cis*-acting elements. ABRE: ABA responsive element; ARE: Anaerobic- responsive element; DRE: Dehydration- responsive element; JERE: Jasmonate and/or elicitor responsive element; LTR: Low temperature- responsive element; MBS: MYB-binding site; MRS: MYB recognition site; MYB: v-myb avian myeloblastosis viral oncogene homolog; MYC: Myelocytomatosis; WRE3: Wound response element3

In order to explore the potential function of SoBSKs, *cis*-acting elements in seven *SoBSK* promoter regions were predicted, and the prevalence distribution of *cis*-acting elements for stress- and hormone- responses were schematically depicted (Fig. 4A). A total of 294 *cis*-acting elements were found in *SoBSKs* involved in diverse abiotic stresses, such as light (80 elements), drought (74), heat (29), anaerobism (22), wound (11), cold (2), and phytohormone (76) (Fig. 4B and Additional file 4). Among them, the elements in response to drought (i.e. DRE, MYB, MRS, MYC, and MBS), light (e.g., Box 4, G-box, and GT1-motif), heat (i.e. CCAAT-box, STRE, and AT-rich), and phytohormone accounted for the main parts in each *SoBSK*, and some of these elements were conserved across seven *SoBSKs*, which implied that *SoBSKs* could play important roles in response to drought, light, heat, and various hormones. Importantly, 76 hormone- responsive elements included 28 for ABA (i.e. ABRE), 23 for methyl jasmonate (MeJA) (i.e. TGACG-motif, CGTCA-motif, and JERE), nine for salicylic acid (SA) (i.e. TCA-element), eight for ethylene (i.e. ERE), three for gibberellin acid (GA) (i.e. P-box and TATC-box), and five for auxin (i.e. TGA-element and AuxRR-core) (Fig. 4B). Collectively, these conserved elements in the promoter region suggested that *SoBSKs* were pivotal for these abiotic stresses and phytohormone stimuli.

### Localization of SoBSK1 and SoBSK6 proteins

**Fig. 5:**
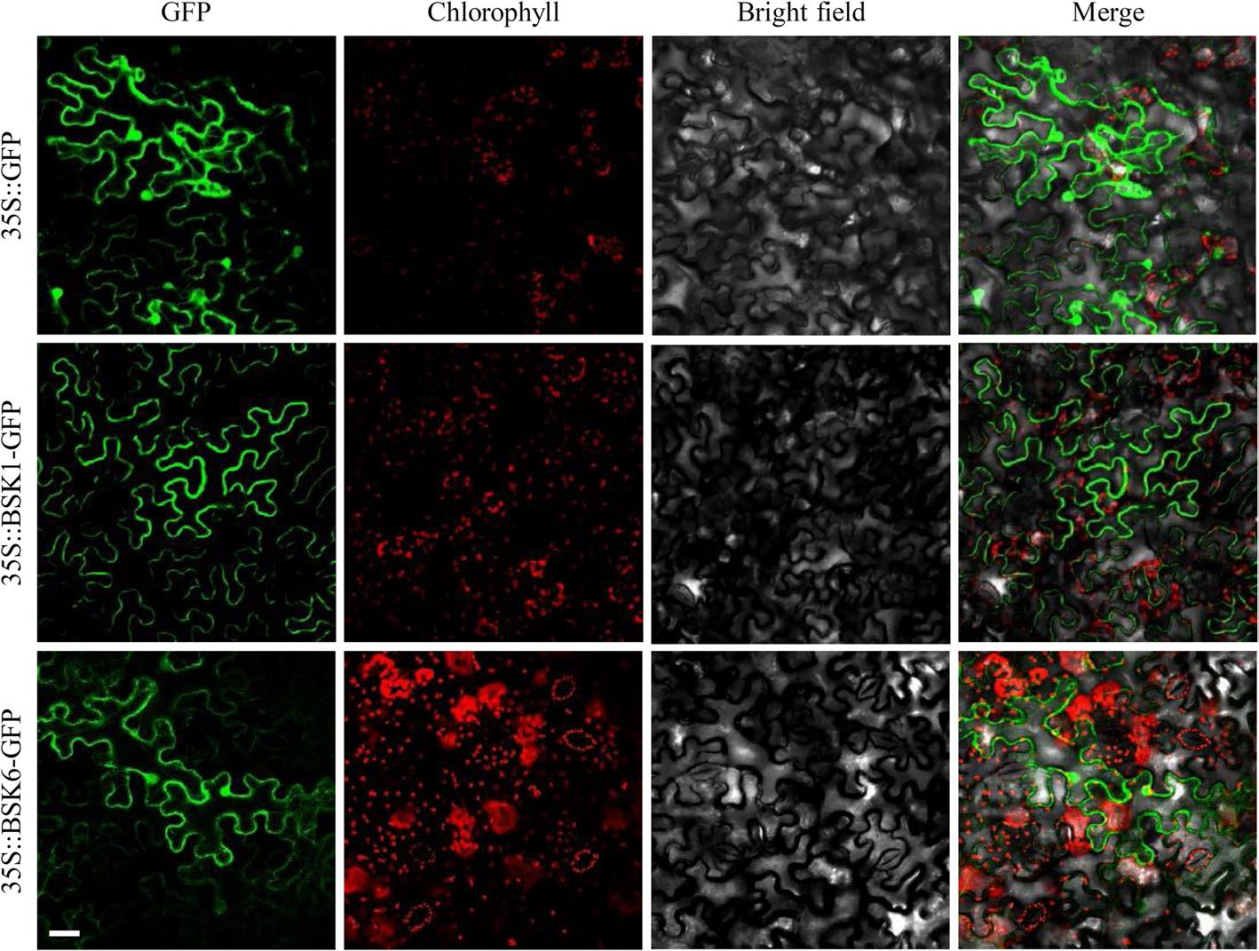
Subcellular localization of SoBSK1 and SoBSK6 in tobacco (*Nicotiana benthamiana*) leaves. 35S::GFP indicated the vector control. All proteins were transiently expressed in epidermal cells of tobacco leaves using *Agrobacterium tumefaciens*, and the GFP signal was visualized after 48 h of infection. Bar = 40 μm

The subcellular localization of SoBSKs was performed using tobacco leaves. *SoBSK1* and *SoBSK6* were fused respectively in frame to the 3’-terminus of the GFP reporter gene under the control of CaMV 35S promoter. The recombinant SoBSK1-GFP, SoBSK6-GFP, and GFP alone were transiently expressed in the epidermal cells of tobacco leaves. The 35S::GFP signals were distributed in the cytosol, nucleus, and PM in tobacco epidermal cells, while the signal from 35S::SoBSK1-GFP was concentrated in the PM (Fig. 5). This suggested that the SoBSK1-GFP recombinant protein localized on the cell membrane. Interestingly, unlike SoBSK1-GFP, SoBSK6-GFP recombinant protein localized not only in the cell membrane, but also in the cytoplasm and nucleus, because SoBSK6 was lack of the N-terminal myristoylation site for potential membrane localization [45]. This indicated that the truncated N-terminus without myristoylation site in SoBSK6 led to the missing of exclusive localization to cell periphery.

### Organ- specific expression analysis of *SoBSKs*

**Fig. 6:**
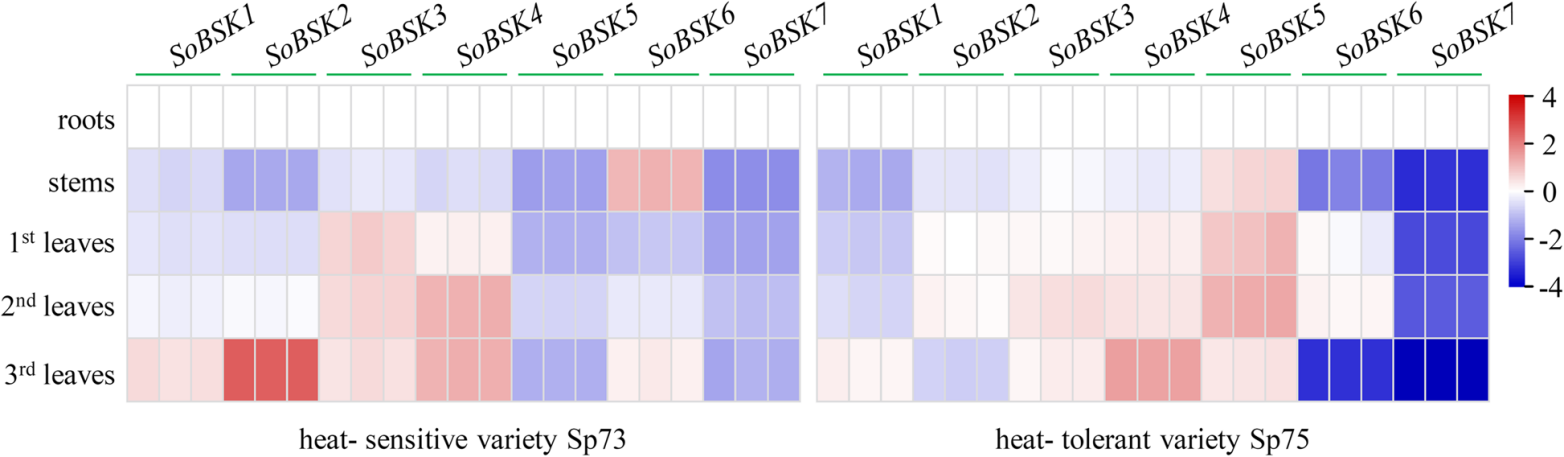
Organ- specific expression analyses of *SoBSKs* in two spinach varieties. Organ specific profiles of *SoBSKs* were analyzed in roots, stems, and leaves from spinach heat- sensitive variety Sp73 and heat- tolerant variety Sp75 using RT-qPCR analysis. The results were calculated via the 2^-ΔΔCt^ method, and the reference gene (*SoARF*) was used to correct the expression levels of *SoBSKs*. The heatmap of *SoBSKs* expression based on the RT-qPCR data standardized by log2 conversion. The color bar on the right of the heat map was based on the RT-qPCR data. Red and blue colors indicated up- and down- regulated genes, respectively

To better understand organ- specific expression of seven *SoBSK* genes, total RNA from roots, stems, and three pairs of alternate leaves (i.e. the 1st, 2nd, and 3rd leaves) from heat- sensitive variety Sp73 and heat- tolerant variety Sp75 were prepared. The transcription level of each *SoBSK* gene was evaluated using reverse transcription quantitative real-time polymerase chain reaction (RT-qPCR) analysis (Fig. 6 and Additional file 5). In heat- sensitive variety Sp73, *SoBSK6* in stems, *SoBSK3* in the 1st leaves, *SoBSK4* in the 2nd leaves, and *SoBSK2* in the 3rd leaves had the highest expression levels (Fig. 6). However, in heat- tolerant variety Sp75, *SoBSK5* in stems, the 1st and 2nd leaves, as well as *SoBSK4* in the 3rd leaves exhibited the highest expressions (Fig. 6). On the other hand, for each gene in variety Sp73, *SoBSK1*, *SoBSK2*, and *SoBSK4* in the 3rd leaves, *SoBSK3* in the 1st leaves, *SoBSK5* and *SoBSK7* in the roots, as well as *SoBSK6* in the stems exhibited the highest expression (Fig. 6). While, in variety Sp75, it was *SoBSK1* and *SoBSK4* in the 3rd leaves, *SoBSK2*, *SoBSK3*, *SoBSK5*, and *SoBSK6* in the 2nd leaves, as well as *SoBSK7* in the roots that had the highest expression (Fig. 6). These suggested that, except of *SoBSK7* in roots, the other *SoBSKs* mainly functioned in leaves rather than stems and roots in different varieties of spinach (Fig. 6).

### ABA- and brassinolide (BL)- responses of *SoBSKs* in leaves

**Fig. 7:**
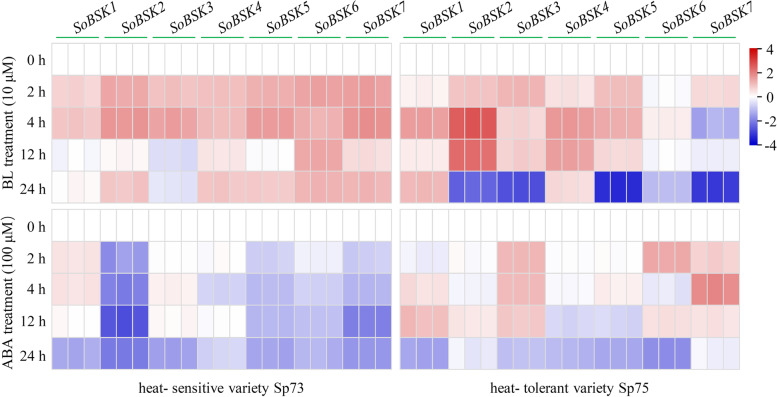
BL- and ABA- responsive expression profiles of seven *SoBSK*s in leaves from spinach heat- sensitive variety Sp73 and heat- tolerant variety Sp75. The data represented the expression levels of *SoBSK*s after BL or ABA treatments for 0, 2, 4, 12, and 24 h, respectively. The results were calculated via the 2^-ΔΔCt^ method, and the *SoARF* was used reference gene to correct the expression levels of *SoBSKs*. The heatmap of *SoBSKs* expression based on the RT-qPCR data standardized by log2 conversion. The color bar on the right of the heat map was based on the RT-qPCR data. Red and blue colors indicated up- and down- regulated genes, respectively

BSKs are critical components in BR signaling pathways [12]. We found multiple *SoBSKs* had ABRE *cis*-acting elements in their promoters for ABA signaling (Fig. 4). This implied that the cross-talk of BR and ABA signaling would happen in regulating plant growth, development, and stress response [46, 47]. Therefore, we evaluated the expression profiles of seven *SoBSKs* in leaves under exogenous BL and ABA treatments using RT-qPCR analysis (Fig. 7 and Additonal file 5).

For BL treatment, all the *SoBSKs* in variety Sp73 were induced more than 1.5- fold at 2 and 4 h. *SoBSK1*, *SoBSK2*, *SoBSK3*, *SoBSK4*, *SoBSK5*, and *SoBSK7* reached their highest expression levels at 4 h, while *SoBSK6* maintained a higher level with more than 2- fold during whole process (Fig. 7). Except of *SoBSK1* and *SoBSK3*, the other *SoBSKs* in variety Sp73 were increased at 24 h (Fig. 7)*.* However, in variety Sp75, *SoBSK2*, *SoBSK3*, and *SoBSK5* were apparently induced at 2, 4, and 12 h, but reduced at 24 h (Fig. 7). Besides, *SoBSK1* and *SoBSK4* were increased, but *SoBSK6* and *SoBSK7* were decreased in variety Sp75 under most conditions (Fig. 7). These indicated that, although the seven *SoBSKs* were all involved in BL- response, most *SoBSKs*, except of *SoBSK6* and *SoBSK7*, were BL- induced in both Sp73 and Sp75 at the early stages (2 and 4 h), and most *SoBSKs* exhibited significantly different even opposite responses in Sp73 and Sp75 under 24 h of BL treatment (Fig. 7). Interestingly, no *SoBSKs* were obviously ABA- induced in Sp73, and most of them were reduced in leaves under various exogenous ABA treatment conditions (Fig. 7). On the contrary, in Sp75, most *SoBSKs* in leaves were ABA- induced at 2 and 4 h, but ABA- reduced at 24 h (Fig. 7). All these suggested that heat- sensitive variety Sp73 and heat- tolerant variety Sp75 probably employed different *BSK*- mediated mechanisms in response to exogenous BR and ABA treatments.

### Temperature stress- response of *SoBSKs* in leaves

**Fig. 8:**
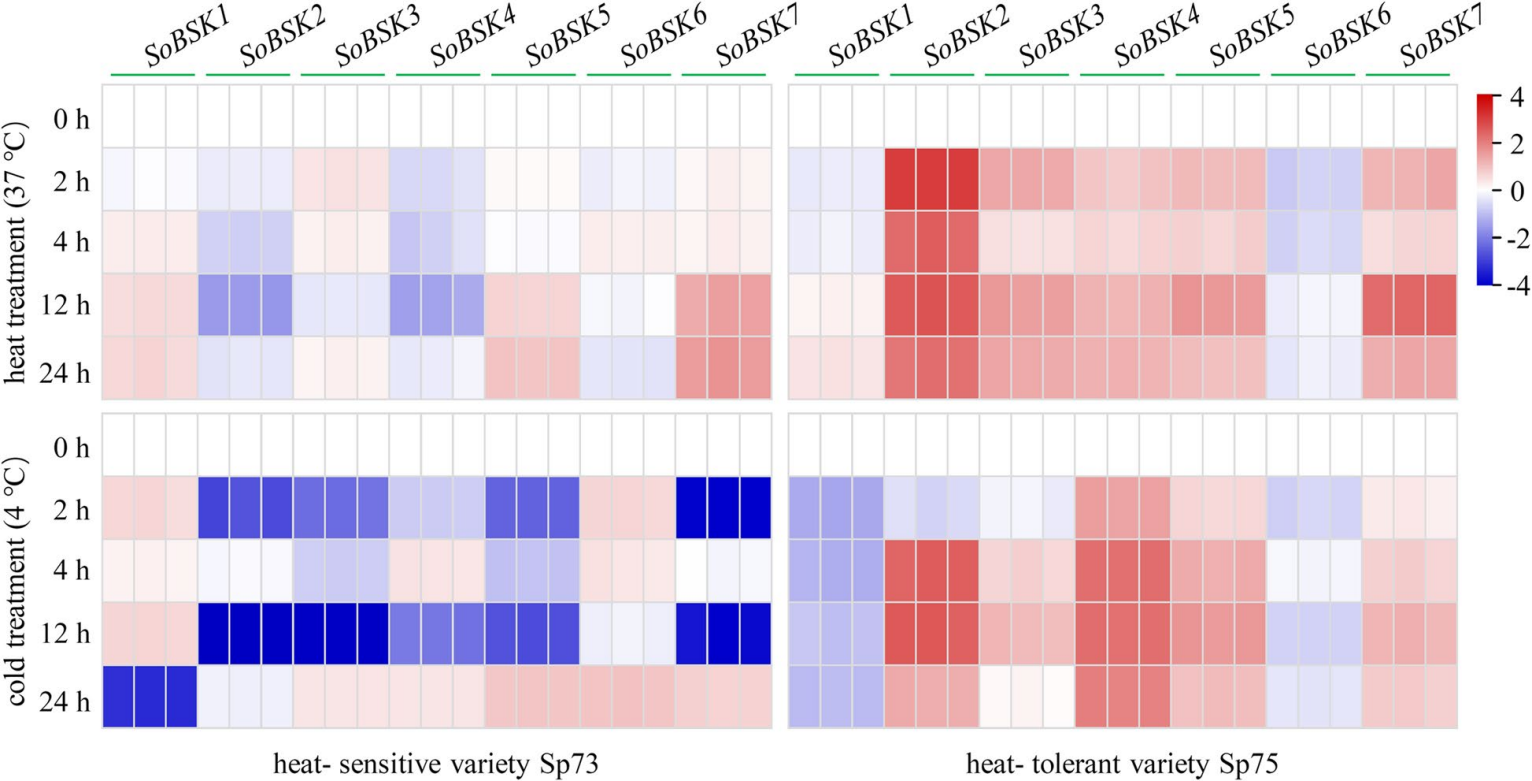
Temperature stress- responsive expression profiles of *SoBSKs* in leaves from spinach heat- sensitive variety Sp73 and heat- tolerant variety Sp75. The data represented the expression levels of *SoBSK*s after heat (37 °C) or cold (4 °C) treatments for 0, 2, 4, 12, 24 h, respectively. The results were calculated via the 2^-ΔΔCt^ method, and the *SoARF* was used as reference gene to correct the expression levels of *SoBSKs*. The heatmap of *SoBSKs* expression based on the RT-qPCR data standardized by log2 conversion. The color bar on the right of the heat map was based on the RT-qPCR data. Red and blue colors indicated up- and down- regulated genes, respectively

Temperature extremes inhibit seed germination and reduce plant growth and reproduction [48]. We have predicted that 29 heat- responsive *cis*-acting elements and two cold- responsive *cis*-acting elements existed in *SoBSKs* promoters (Fig. 4). To evaluate the function of these *SoBSKs* in temperature response, the expression profiles of *SoBSK* genes in the heat- sensitive variety Sp73 and heat- tolerant variety Sp75 under heat and cold stresses were validated using RT-qPCR analysis. Most *SoBSKs* were significantly altered in both varieties under certain temperature stress condition when compared with the normal condition (0 h) (Fig. 8 and Additional file 5). Importantly, the opposite temperature- responsive expression patterns of most *SoBSKs* were observed in varieties of Sp73 and Sp75. Five *SoBSKs*, except of *SoBSK1* and *SoBSK6*, were reduced in Sp73, but induced in Sp75 at 4 and 12 h of cold and heat stresses (Fig. 8). Under heat conditions, *SoBSK2* and *SoBSK4* were obviously decreased in Sp73, but *SoBSK5* and *SoBSK7* were increased at 12 and 24 h. However, five *SoBSKs* (i.e. *SoBSK2*, *SoBSK3*, *SoBSK4*, *SoBSK5*, and *SoBSK7*) were heat- induced in Sp75 under each time point, which was represented by the significantly heat- induced *SoBSK2* (Fig. 8). The similar expression patterns were found under cold conditions. Five *SoBSKs* (i.e. *SoBSK2*, *SoBSK3*, *SoBSK4*, *SoBSK5*, and *SoBSK7*) were cold- increased in Sp75, but cold- decreased in Sp73, while two *SoBSKs* (i.e. *SoBSK1* and *SoBSK6*) were slightly induced in Sp73, but reduced in Sp75 (Fig. 8).

### Heat- responsive genes in BR- and ABA- signaling in leaves

**Fig. 9:**
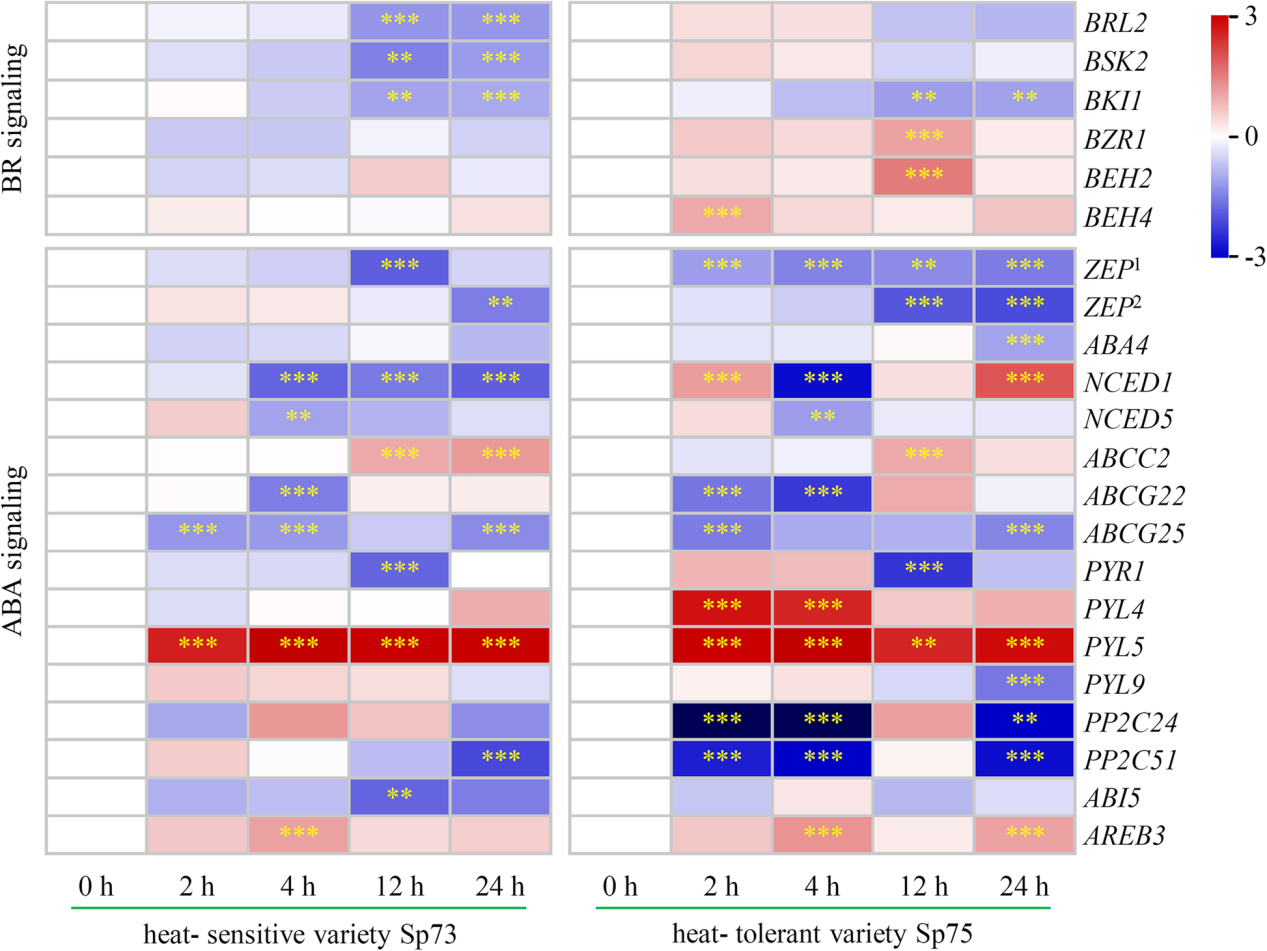
Differentially expressed genes in BR and ABA signaling in spinach under heat stress revealed from transcriptomic analysis. The columns represented fold change (Log2 transformed) of genes in leaves under heat treatment for 2, 4, 12, and 24 h compared with 0 h. The rows represented individual genes, and the genes with various Gene ID were marked with different numbers companied with Gene symbols. The color bar on the right of the heat map was based on the transcriptome data. Red and blue colors indicated up- and down- regulated genes, respectively. **, *p* < 0.01; ***, *p* < 0.001. The detailed information was included in Additional file 6. ABA4: ABA deficient4; ABCC: ABC transporter C family member; ABCG: ABC transporter G family member; ABI5: ABSCISIC ACID-INSENSITIVE 5; AREB3: ABRE binding factor3; BEH: bri1 EMS SUPPRESSOR1/ BRASSINAZOLE RESISTANT1 homolog protein; BES1: bri1 EMS SUPPRESSOR1; BKI1: BRI1 kinase inhibitor 1; BRL2: BRI1-like 2; BSK2: BR SIGNALING KINASE2; BZR1: BRASSINAZOLE RESISTANT1; NCED: 9-*cis*-epoxycarotenoid dioxygenase; PP2C: Protein phosphatase 2C; PYL: PYRABACTIN RESISTANCE 1-LIKE; PYR: PYRABACTIN RESISTANCE; ZEP: Zeaxanthin epoxidase

The expression levels of six BR signaling- related genes and 16 ABA signaling- related genes were detected in leaves from variety Sp73 and variety Sp75 under heat treatments for 0, 2, 4, 12, and 24 h using RNA-sequencing assay (Fig. 9 and Additional file 6). Two genes (*BRL2* and *BSK2*) for BR signal perception were slightly heat- induced in variety Sp75 at 2 and 4 h, but significantly decreased in variety Sp73 at 12 and 24 h. However, the *BKI1* for inhibition of BR binding with BRI1 was heat- reduced in Sp73 and Sp75 at 12 and 24 h (Fig. 9). Besides, three genes encoding transcription factors in the BR- mediated signaling pathway, including *BZR1*, *BEH2*, and *BEH4*, were induced in variety Sp75 under heat treatment (Fig. 9). This implied that BR signaling was probably employed in variety Sp75 for facilitating its heat tolerance.

The other 16 heat- altered genes were involved in ABA biosynthesis, transport, signal transduction, and transcriptional regulation (Fig. 9). Five genes for ABA biosynthesis, including two *ZEPs*, *ABA4*, *NCED1*, and *NCED5*, were reduced in variety Sp73, and four of them were also reduced in variety Sp75, except of *NCED1* induced at 2 and 24 h (Fig. 9). Besides, *ABCC2* for ABA transport from cytoplasm to vacuole was induced, but ABA efflux transporter *ABCG25* and influx transporter *ABCG22* for long-distance ABA transport were reduced in both Sp73 and Sp75 (Fig. 9). These indicated that the biosynthesis and transport of ABA were heat- disturbed in spinach, while some specific pathways probably were enhanced in heat- tolerant variety Sp75. Significantly, the expressions of four ABA receptor encoding genes (i.e. *PYR1*, *PYL4*, *PYL5*, and *PYL9*) were changed under heat treatments. Among them, *PYL5* was significantly heat- induced in variety Sp73 and Sp75, and *PYL4* was induced in Sp75 at 2 and 4 h (Fig. 9). Interestingly, *PP2C24* and *PP2C51*, the negative regulation factor of ABA signaling, were obviously reduced in Sp75 under heat stress. Besides, the expressions of transcription factors *ABI5* and *AREB3* were altered, and *AREB3* was increased in both Sp73 and Sp75 under heat treatments (Fig. 9). All these implied that variety Sp75 exhibited more enhanced ABA signaling to regulate heat- responsive process when compared with variety Sp73.

### Heat-responsive phenotype of *SoBSK1*- overexpressing Arabidopsis plants

**Fig. 10:**
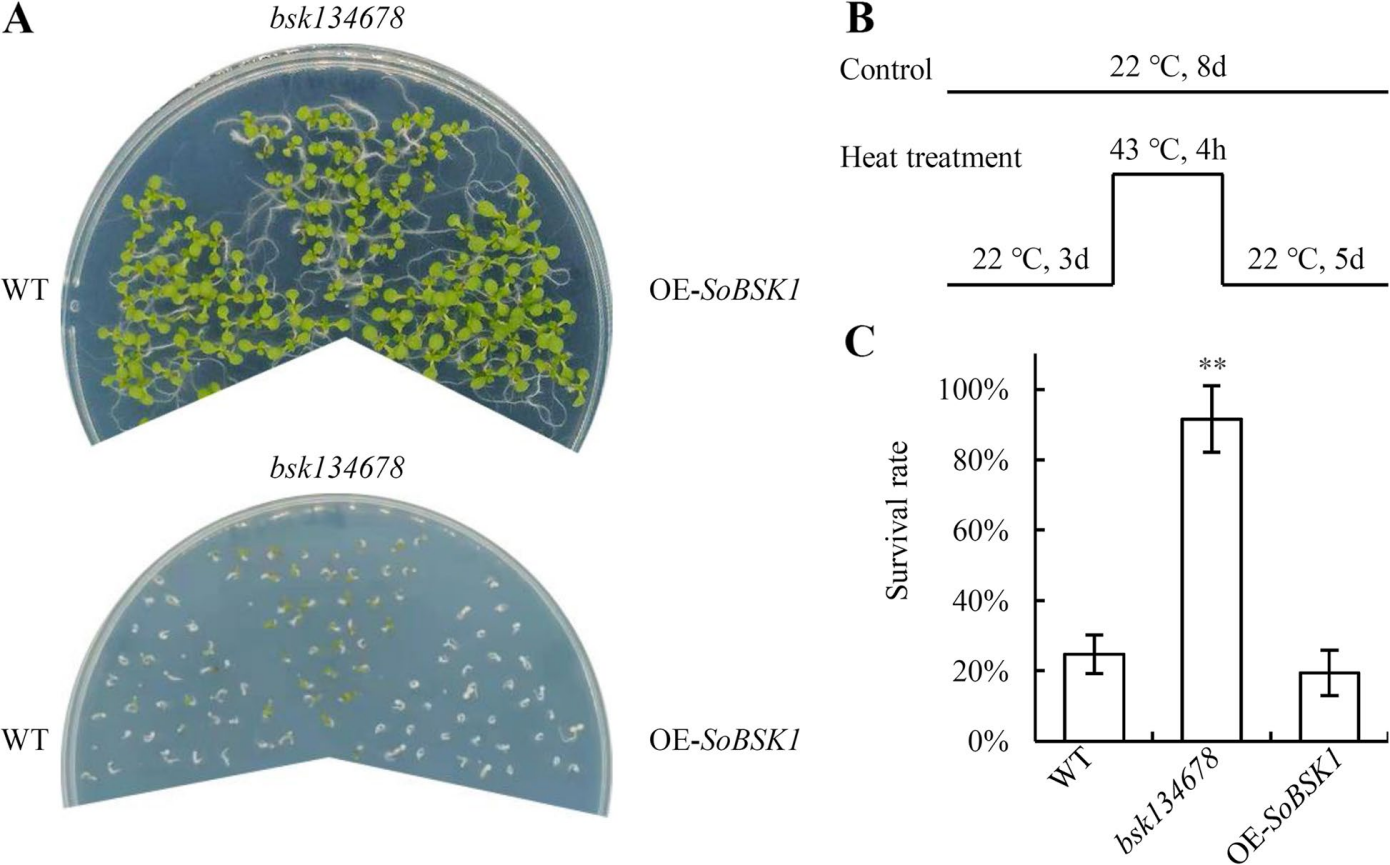
Thermotolerance analysis of the wild- type (WT), *bsk134678* mutant, and *SoBSK1*- overexpressed *A. thaliana* seedlings. **A** Phenotypes of WT, *bsk134678* mutant, and *SoBSK1*- overexpressed *A. thaliana* seedlings grown on 1/2 MS medium under 22 °C or heat treatment for eight days. **B** Schematic diagram of plant treatment conditions. The seedlings grown under 22 °C for eight days were control. The heat- treated seedlings were grown under 22 °C for three days, then 43 °C for 4 h, and following under 22 °C for five days. **C** Survival rate of seedlings from WT, *bsk134678* mutant, and *SoBSK1*- overexpressed *A. thaliana* after heat treatment. Each bar indicated the mean ± standard deviation (SD) (*n* = 3, **P < 0.01)

The *SoBSK1* expression pattern was distinct with other *SoBSKs* in heat- tolerant variety Sp75 in response to heat treatment (Fig. 8). To investigate the function of *SoBSK1* on heat tolerance, we constructed overexpressing *SoBSK1* transgenic Arabidopsis plants under the control of the strong constitutive CaMV 35S promoter, and compared the survival rates of wild- type (WT), *bsk134678* mutant, and *SoBSK1*- overexpressed seedlings under heat treatment (Fig. 10). Three- day- old WT, *bsk134678* mutant, and *SoBSK1*- overexpressed seedlings grew under 22 °C for five days as control, or treated under 43 °C for 4 h, and following under 22 °C for five days as heat treatments (Fig. 10A and B). There were no differences among WT, *bsk134678* mutant, and *SoBSK1*- overexpressed seedlings under control condition, but they exhibited obvious different phenotypes under heat treatments (Fig. 10A). The *bsk134678* mutant seedlings showed higher survival rate (92%), which was about 3.7- fold and 4.7- fold higher than WT and *SoBSK1*- overexpressed seedlings, respectively (Fig. 10A and C). While WT and *SoBSK1*- overexpressed seedlings exhibited similar phenotype, and no significantly different survival rate was observed (Fig. 10C). These implied that *SoBSK1* probably regulated plant heat tolerance as a negative factor and the members of BSK family had function redundance.

## Discussion

### The SoBSKs had close homology with sugar beet BSKs

The BSKs belonged to the receptor-like cytoplasmic kinase superfamily RLCK-XII, which were firstly identified in *A. thaliana* [11]. Recently, the genome- wide identification of 143 BSKs from 17 representative plant species in different evolution positions has been reported [17]. Besides, the expression patterns of some members of *BSKs* were investigated in wild barley [36], Kentucky bluegrass [37], and *P. tomentosa* [38] in response to drought and cold, respectively. However, only a few of *BSKs* were cloned and characterized in plants, such as *AtBSK1*, *AtBSK2*, *AtBSK3*, and *AtBSK5* in Arabidopsis [11, 18, 21–30, 33, 40], *OsBSK1*–2 and *OsBSK3* in rice [15, 16], *NsBSK1* and *NsBSK3* in woodland tobacco [42], *NtBSK2* in common tobacco [42], and *ZmBSK1* in maize [35]. The phylogenetic and functional analyses of *BSK*s from green vegetables, such as spinach, have not been reported so far.

In this study, seven *SoBSK* genes were identified in the genome and cloned from spinach variety Sp75. These SoBSKs, together with other 65 BSKs from Arabidopsis, sugar beet, common tobacco, woodland tobacco, rice, and maize, were grouped into seven classes in the phylogenetic tree (Fig. 1). Our results indicated that seven SoBSKs were closely homologous with BvBSKs from sugar beet, which was consistent with the results of whole- genome comparative analysis [1]. Sugar beet was another Caryophyllales species, which genome was relatively smaller (760 Mb) and closely related to spinach [1]. Moreover, the predicted number of protein-coding genes (25,495), the average length of the coding sequences (1157 bp), the number of average exons (5.3), and the number of transcription factors (1202) in spinach genome were all similar with those in sugar beet genome [1]. Therefore, BSK was a well- representative gene family revealing the close relationship between spinach and sugar beet.

In addition, our results indicated that the BSKs from dicotyledons (i.e. spinach, sugar beet, Arabidopsis, common tobacco, and woodland tobacco) and monocotyledons (i.e. rice and maize) were classed into separated subgroups in each class (Fig. 1), implying the lineage- specific evolution of *BSK* genes after the divergence of dicots and monocots. This was also found in the analysis of the genome- wide identification of 143 BSKs from 17 plant species, although spinach, common tobacco, woodland tobacco, and sugar beet were not included [17]. Interestingly, the expansion of *BSK* gene family attributed to the whole genome duplication (WGD) in some of the previously reported 17 plant species, such as two- fold expansion in Arabidopsis (12 *BSKs*), soybean (*Glycine max*) (13 *BSKs*), and *Populus trichocarpa* (14 *BSKs*), as well as three- fold expansion in *Brassica rapa* (21 *BSKs*) [17], but the WGD events were not observed in spinach (7 *BSKs*) [1]. Gene family members can be extended by duplication events under evolution processes [49]. It seems that the duplication events might have happened before species divergence in spinach.

### SoBSKs had conserved TPR domain and kinase domain

Seven SoBSKs had conserved TPR domain at the C-terminus, including two TPRs in SoBSK2 and SoBSK3, and three TPRs in the other five SoBSKs (Fig. 3G). The conserved TPRs also existed in the BSKs from Arabidopsis [17] and rice [16]. TPR domain functioned to facilitate the specific interaction between partners acting as scaffolds for the assembly of multiprotein complexes [21, 50]. The TPR in rice OsBSK3 was proved to an autoregulatory domain as a “phospho-switch” for modulating BSK kinase activity [16]. The TPR bound to the kinase domain of BSK3 for the prevention of BSK3 from binding to and activating BSU1 without BR signals, while the phosphorylated kinase domain of BSK3 by BR- activated BRI1 was free from the binding with the TPR domain, and then facilitated the binding affinity of BSKs for downstream phosphatase BSU1 to promote BR signaling pathway [16]. It has been found that TPR domain was critical for several BSK’s functions in embryogenesis and defense responses [16, 26, 51]. In Arabidopsis, the missense mutation of the strictly conserved arginine (R) site R^443^ (R443Q) in the TPR domain led to the loss-of-function of AtBSK1, conferring the defective defense responses [26]. Moreover, the TPR deletion of SSP/ BSK12 completely inactivated its function, resulting in the defective suspensor development upon Arabidopsis embryogenesis [51]. In rice, the TPR domain negatively regulated OsBSK3 activity in BR signaling [16]. On the contrary, the truncated AtBSK3 protein without TPR motifs (BSK3^TPR-Δ^) was less efficient to rescue the Arabidopsis root growth defect and BR resistance phenotype of *bsk3–1* when compared with BSK3 protein, although it can fully complement the *bsk3–1* mutant phenotypes. This indicated that TPR domain was not essential, but required for the full function of AtBSK3 in BR signaling [21]. Additionally, TPR deletion impaired the interactions of AtBSK3-AtBSK3 and AtBSK3-AtBSK1, implying its regulation to BSK homodimer and/ or heterodimer formation [21]. Interestingly, all the seven SoBSKs had the conserved TPR domain and R residue inside (Fig. 3G), which functions remained to be elucidated.

All SoBSKs contained conserved phosphorylation site of serine in the kinase domain, except of SoBSK6 (Fig. 3G). Their homologous serine sites in AtBSKs and OsBSKs were also conserved and critical for their kinase functions [11, 16, 26, 41]. The S^230^ in AtBSK1, S^210^ in AtBSK3, S^209^ and T^210^ in AtBSK5, S^215^ in OsBSK3, as well as the adjacent S, T, and Tyrosine (Y) residues in AtBSK6 and AtBSK8 have been reported to be phosphorylated by BRI1, RLK902, PEPR1, and ELONGATION FACTOR-TU RECEPTOR (EFR) and functioned in the BR signaling pathway upon plant immunity, respectively [11, 16, 18, 29]. Phosphorylation events as a PTM can cause the critical changes in stability and activity of target proteins as well as protein-protein interactions [52, 53]. Therefore, these conserved phosphorylation sites in SoBSKs would be pivotal for their functions in spinach development and stress response. Additionally, in the kinase domain, a lysine in six SoBSKs except of SoBSK6 were conserved, and two lysines (K^100^ in SoBSK1 and K^104^ in SoBSK5) were predicted to be ubiquitinated (Fig. 3G). The conserved lysine in six SoBSKs were homologous with K^104^ in AtBSK1 [26], K^86^ in AtBSK3 and AtBSK5 [18, 21], as well as K^87^ in AtBSK8 [54], which have been proved to the key sites of ATP binding for BSK kinase activity. However, the regulation role of this ubiquitination site in kinase domain for balancing the BSK degradation and its kinase activity needs to be investigated. Additionally, the conserved glycine sites in all SoBSKs (Fig. 3G) were homologous with G^238^ and G^226^, which were found to interfere with the protein stability of AtBSK3 and other BSKs in BR signaling, respectively [21].

### SoBSKs played important roles in spinach development and heat response

BSKs represent a critical kinase family that play partially redundant or overlapping roles in BR signaling and/ or other kinase signaling pathways during plant development and stress response [11, 20]. In spinach, different *SoBSK* members displayed a constitutive and unique expression pattern in roots, stems, and leaves from variety Sp73 and variety Sp75, and most of them had higher expression in developing leaves (i.e. the 3rd and 2nd leaves) than that in the mature leaves (the 1st leaves) (Fig. 6), which implied *SoBSKs* were important for spinach growth and development. The similar expression patterns of *BSKs* were also found in Arabidopsis. Twelve *AtBSKs* were expressed in multiple tissues and organs (i.e. roots, flowers, rosette leaves, mature pollen, and seeds) during various developmental stages [17]. Phenotype analyses of various single, double, and multiple mutations of each AtBSK indicated that BSKs were partially redundant positive regulators of BR signaling in Arabidopsis [20]. Besides, the complex genetic interactions between BSKs (e.g., homodimers and/ or heterodimers) and their downstream proteins were supposed to the possible reasons for distinct functional characteristics of BSKs in diverse physiological and developmental processes [20].

In our results, most *SoBSKs* were BL- induced in leaves from both heat- sensitive variety Sp73 and heat- tolerant variety Sp75 (Fig. 7), which indicated that most *SoBSKs* were involved in the BR signaling pathway, and 2 to 4 h of BL treatments would be the critical time point for BR signal perception and original transduction in two spinach varieties. Importantly, several *SoBSKs* (i.e. *SoBSK2*, *SoBSK5*, *SoBSK6*, and *SoBSK7*) were significantly ABA- induced in leaves from heat- tolerant variety Sp75, but ABA- reduced in heat- sensitive variety Sp73 under certain time points (Fig. 7). Moreover, *SoBSK1* and *SoBSK3* were highly heat- induced in variety Sp75 when compared with that in variety Sp73 (Fig. 7). This implied that heat- tolerant variety Sp75 potentially had stronger stress tolerance depending on ABA signaling pathway than heat- sensitive variety Sp73. Consistently, some *AtBSKs* were also significantly altered in response to various exogenous hormones (i.e., BL, ABA, JA, GA-3, Auxin, Zeatin, and 1-aminocyclopropane-1-carboxylic acid (ACC, ethylene precursor)) [17], implying that *BSKs*- mediated pathways had cross-talks with other hormone signaling pathways.

Importantly, our results indicated that the expression patterns of *SoBSKs* in leaves were altered under certain heat- and/ or cold- stress conditions, and most *SoBSKs* increased obviously in heat- tolerant variety Sp75 than those in heat- sensitive variety Sp73 (Fig. 8). Taken together with the ABA and drought responses of *BSKs* from Arabidopsis (i.e. *AtBSK3*, *AtBSK5*, and *AtBSK8*) and maize (*ZmBSK1*) [17, 24, 35], we supposed that *BSKs* were involved in the heat response via regulation of ABA- mediated stomatal closure and ROS homeostasis [24, 55]. Thermotolerance assay of WT, *bsk134678* mutant, and *SoBSK1*- overexpressed Arabidopsis seedlings suggested that *SoBSKs* participated in response to heat treatments redundantly, and *SoBSK1* could be a negative regulator under heat conditions (Fig. 10). Besides, their homologous *AtBSKs* were also significantly altered under heat and cold conditions. Most *AtBSKs* were reduced under heat stress, but *AtBSK1*, *AtBSK2*, and *AtBSK9* were induced in Arabidopsis under cold stress after 24 h [17]. Similarly, in *Populus tomentosa*, *PtBSK*, *PtBRI1*, and *PtBIN2* were all increased under cold stress [38].

Additionally, the BSKs- mediated cross-talks of BR- signaling pathway and other pathways (e.g., ABA signaling, auxin signaling, ROS signaling, and MAPK cassette) were reported to function in diverse environment responses, such as salts [24, 25, 47, 56, 57], drought [17, 35], low nitrogen [22], exogenous sucrose supply [41], as well as oxidative stress and wounding [17]. In this study, we evaluated the important gene expression in BR signaling and ABA signaling in spinach varieties Sp75 and Sp73 under heat stress using RNA-seq analysis. Our results indicated that heat- tolerant variety Sp75 was likely to improve heat resistance by promoting BR- mediated downstream pathways through BR signaling transcription factors (i.e. *BZR1*, *BEH2*, and *BEH4*), and activating ABA signaling through regulating *PYL4* and *PP2Cs* (Fig. 9). However, the key members and functions of BSKs in these fine-tuned modulation mechanisms, as well as the cross-talk of BR and ABA signaling still need to be investigated.

## Materials and methods

### Identification and cloning of *BSK* genes in spinach

To identify the BSK family in spinach, the genome was downloaded from the SpinachBase (http://www.spinachbase.org/). We used 12 AtBSK protein sequences as queries to search against the spinach genome database using BLASTp with an expected value (*e*-value) cutoff of 1E^− 100^. Candidate SoBSKs were submitted to the NCBI web CD-search tool (https://www.ncbi.nlm.nih.gov/Structure/bwrpsb/bwrpsb.cgi) to verify their conserved kinase domain and TPR domain. To verify the accuracy of the predicted *BSK* sequences in spinach genome database, the coding sequences (CDSs) of *BSKs* were cloned with the *SoBSKs* primers (Additional file 7). The CDSs and protein sequences of SoBSKs were shown in Additional file 1.

### Phylogenetic analysis of BSKs from different species

The phylogenetic tree of BSKs in spinach and other six plant species (i.e. sugar beet, Arabidopsis, common tobacco, woodland tobacco, maize, and rice) were constructed with MEGA-X by the neighbor- joining (NJ) method and 1000 bootstrap replicates. Herein, 12 AtBSKs [11], five OsBSKs [15, 16], two NsBSKs [42], one NtBSK [42], and nine ZmBSKs [35] were reported previously, and all the other protein sequences of BSKs were obtained by BLSATp with AtBSKs as queries to search against the genome databases, including RefBeet-1.2.2 (https://bvseq.boku.ac.at/index.shtml), Ntab-TN90 (https://www.ncbi.nlm.nih.gov/data-hub/assembly/GCF_000715135.1/), Nsyl (https://www.ncbi.nlm.nih.gov/data-hub/assembly/GCF_000393655.1/), and Zm-B73-REFERENCE-NAM-5.0 (https://www.ncbi.nlm.nih.gov/data-hub/assembly/GCF_902167145.1/) for sugar beet, common tobacco, woodland tobacco, and maize, respectively.

### Chromosomal distribution and collinearity analysis of *SoBSK* genes

The position information (e.g., chromosomal distribution, length, as well as the start and end positions) of *SoBSK* genes on the chromosome were obtained from SpinachBase, and visualized by MapGene2Chrom web v2 (http://mg2c.iask.in/mg2c_v2.0/). The collinearity analysis of *BSKs* between the homologs in spinach and Arabidopsis, sugar beet, or maize were verified and visualized using One Stem MCScanX and Dual Systeny Plot for MCScanX in TBtools software, respectively.

### Analyses of gene structure, function domain, conserved motif, and PTM

The exon-intron structure was analyzed based on the full-length genome sequences and the CDSs of *SoBSKs* by TBtools. Functional domains of SoBSKs were detected by the NCBI web CD- search tool. The conserved motifs of SoBSKs were determined by MEME (https://meme-suite.org/meme/tools/meme) using the protein sequences with the maximum motif number of 13, and were visualized by TBtools. The conserved phosphorylation, myristoylation, and reported conserved functional sites in SoBSKs were analyzed by sequence alignment with previous researches in Arabidopsis [11, 18, 21, 26, 27, 29, 41] and rice [16]. The S-nitrosylation and ubiquitination sites were predicted by GPS-SNO 1.0 [43] with default parameters and UbiComb (http://nsclbio.jbnu.ac.kr/tools/UbiComb/) with a threshold value of 0.5 [44], respectively.

### Identification of putative *cis*-regulatory elements in the promoters

The promoter sequences (2000 bp before the start codon) of all *SoBSKs* were extracted from the SpinachBase, and were analyzed by using the PlantCARE (http://bioinformatics.psb.ugent.be/webtools/plantcare/html/) and visualized by TBtools.

### Plant materials, RNA extraction, cDNA synthesis, and RT-qPCR

The spinach heat- sensitive variety Sp73 and heat- tolerant variety Sp75 were used for gene expression analysis [58]. The seeds were sown in a tray containing a perlite-matrix (1:1) mixture and grown in a plant growth chamber. The growth conditions were set as 22 °C 10 h light/ 18 °C 14 h dark and 60% relative humidity [3]. The expression levels of *SoBSK* genes in various tissues including roots, stems, leaves (i.e. the 1st, 2nd, and 3rd leaves) were detected. Temperature stresses (4 °C for cold and 37 °C for heat) and exogenous hormone treatments (10 μM BL and 100 μM ABA) were applied for 0, 2, 4, 12, and 24 h with six- leaf stage of seedlings, respectively.

Total RNA was extracted from spinach leaves with TRIzol™ LS Reagent (Invitrogen, USA) [59]. The RNA was reverse transcribed into cDNA using the Prime Script RT reagent kit. The RT-qPCR was performed using the SYBR Premix ExTaq II kit (TRAN, China) with *SoARF* as an internal control. Specific primer pairs were designed using Primer3 Web tool (http://bioinfo.ut.ee/primer3/) (Additional file 7). RT-qPCR reactions were performed with three independent biological replicates. Besides, at least three technical replicates for some representative genes were performed in each set of RT-qPCR analyses (Additional file 5). Each gene was normalized to the *SoARF* internal control gene, and the relative gene expression was calculated according to the 2^-ΔΔCt^ method [60].

### Differentially expressed genes (DEGs) analysis by RNA-sequencing

Total RNA was extracted from leaves of the spinach heat- sensitive variety Sp73 and heat- tolerant variety Sp75 after heat treatments at 0, 2, 4, 12, and 24 h using the mirVana miRNA Isolation Kit (Ambion). Three independent biological replicates were performed for RNA-sequencing analysis (Additional file 6). RNA integrity was evaluated using the Agilent 2100 Bioanalyzer (Agilent Technologies, USA). The samples with RNA integrity number (RIN) ≥ 7 were subjected to enrich mRNA and construct cDNA libraries using TruSeq Stranded mRNA LTSample Prep Kit (Illumina, USA) according to the manufacturer’s instructions. The libraries were sequenced on the HiSeq 2500 platform (Illumina, USA). The high- quality clean reads were obtained by removing adaptor sequences, empty reads low- quality bases (Q < 30) and used for transcriptome de novo assembly by mapping to spinach reference genome [1] using hisat2.

Fragments Per Kilobase of transcript per Million (FPKM) of each gene and read counts value of each transcript (protein_coding) were calculated using bowtie2 and eXpress. Differential expression analysis among the samples was performed using the DESeq (2012) R package. The genes with *p* value < 0.05 and Fold Change > 2 (or < 0.5) across the heat- stressed and control (0 h in heat- sensitive variety Sp73 or heat- tolerant variety Sp75) samples in at least two replicates were designated as DEGs in each variety.

### Subcellular localization of SoBSK1 and SoBSK6

The open reading frames (ORFs) of *SoBSK1* and *SoBSK6* without stop codon were introduced into the plant expression vector pCAMBIA2300-GFP to produce SoBSK1-GFP and SoBSK6-GFP fusion proteins under the control of the CaMV 35S promoter. The resulting constructs (i.e. pCAMBIA2300-SoBSK1-GFP and pCAMBIA2300-SoBSK6-GFP) and the empty vector pCAMBIA2300-GFP were introduced into *Agrobacterium tumefaciens* strain EHA105. 35S::SoBSK1-GFP, 35S::SoBSK6-GFP, and 35S::GFP (the vector control) were introduced into six- week- old tobacco leaves by infiltration using needle-less syringes [61]. The instantaneously transformed tobacco leaves grew in dark for 24 h, following under normal conditions (25 °C 16 h light/ 20 °C 8 h dark) for 24 h. The GFP signals in transformed tobacco epidermal cells were visualized under an Olympus FV3000 confocal laser scanning microscope (Olympus, Japan). The GFP green fluorescent signals in 35S::SoBSK1-GFP and 35S::SoBSK6-GFP transformed tobacco epidermal cells, as well as their merged images with the chlorophyll fluorescence and bright field signals were used to determine the localization of SoBSK1 and SoBSK6 in the cell, respectively. The 35S::GFP was used as a control sample that showed the GFP fluorescence in the whole cell [62].

### Thermotolerance analysis of transgenic Arabidopsis

The *A. tumefaciens* strain EHA105 cells containing pCAMBIA2300-SoBSK1-GFP plasmid were transformed into *A. thaliana* WT seedlings using the floral dip method [63] to obtain *SoBSK1*- overexpressed seedlings. The transformed seedlings were screened on 1/2 MS agar medium containing 50 μg/mL kanamycin for three generations. The *SoBSK1* expression levels of several independently homozygous T3 lines were evaluated by RT-qPCR with gene-specific primers (Additional file 7), and the highest expressed line was selected for thermotolerance analysis. WT, *bsk134678* mutant, and *SoBSK1*- overexpressed *A. thaliana* seedlings were grown on 1/2 MS medium in a light incubator (22 °C 16 h light/ 20 °C 8 h dark, and 75% relative humidity) for eight days were control. The heat- treated seedlings were grown under control conditions for three days, then 43 °C for 4 h, and following under control conditions for five days. After treatments, the photographs were taken and the survival rates of seedlings from WT, *bsk134678* mutant, and *SoBSK1*- overexpressed *A. thaliana* were calculated with three biologically independent replicates.

## Supplementary Information

**Additional file 1.**

**Additional file 2.**

**Additional file 3.**

**Additional file 4.**

**Additional file 5.**

**Additional file 6.**

**Additional file 7.**

## Data Availability

The *SoBSK* gene sequences and protein sequences in this study are available in the spinach genome database SpinachBase (http://www.spinachbase.org/). The spinach heat- sensitive variety Sp73 and heat- tolerant variety Sp75 materials used in the experiment were obtained in our laboratory. The datasets generated and/ or analyzed during this current study are included within this article and its additional files.

## Abbreviations

ABA: Abscisic acid
ABA3: ABA deficient3
ABA4: ABA deficient4
ABCC: ABC transporter C family member
ABCG: ABC transporter G family member
ABI5: ABSCISIC ACID-INSENSITIVE 5
ABRE: ABA responsive element
ACC: 1-aminocyclopropane-1-carboxylic acid
AREB3: ABRE binding factor3
BAK1: BRI1-ASSOCIATED RECEPTOR KINASE1
BEH: Bri1 EMS SUPPRESSOR1/BRASSINAZOLE RESISTANT1 homolog protein
BES1: Bri1 EMS SUPPRESSOR1
BIN2: BRASSINOSTEROID INSENSITIVE2
BIR1: BAK1-INTERACTING RECEPTOR-LIKE KINASE1
BKI1: BRI1 kinase inhibitor 1
BL: Brassinolide
BR: Brassinosteroid
BRI1: BR INSENSITIVE1
BRL2: BRI1-like 2
BSK: BR SIGNALING KINASE
BSU1: BRI1 SUPPRESSOR 1
BZR1: BRASSINAZOLE RESISTANT1
CDS: Coding sequence
DEG: Differentially expressed gene
DRE: Dehydration- responsive element
EFR: ELONGATION FACTOR-TU RECEPTOR
ERE: Ethylene responsive element
ETI: Effector-triggered immunity
*e*-value: Expected value
FLS2: FLAGELLIN SENSING2
FPKM: Fragments Per Kilobase of transcript per Million
G: Glycine
GA: Gibberellin acid
GE: Glucosyl ester
JERE: Jasmonate and/or elicitor responsive element
K: Lysine
LYK5: LYSIN-MOTIF RECEPTOR KINASE5
MAPK: Mitogen-activated protein kinase
MAPKKK5: Mitogen-activated protein kinase kinase kinase5
MBS: MYB-binding site
MeJA: Methyl jasmonate
MRS: MYB recognition site
MYB: V-myb avian myeloblastosis viral oncogene homolog
MYC: Myelocytomatosis
NCED: 9-*cis*-epoxycarotenoid dioxygenase
ORF: Open reading frame
PAMP: Pathogen-associated molecular pattern
PEPR1: PEP1 RECEPTOR1
PIF4: BES1-PHYTOCHROME INTERACTING FACTOR4
PM: Plasma membrane
PP2C: Protein phosphatase 2C
PTI: Pathogen-associated molecular pattern- trigged immunity
PTM: Post-translational modification
PYL: PYRABACTIN RESISTANCE 1-LIKE
PYR: PYRABACTIN RESISTANCE
R: Arginine
RIN: RNA integrity number
RLCK: Receptor-like cytoplasmic kinase
RLK902: RECEPTOR-LIKE KINASE 902
ROS: Reactive oxygen species
RPS2: Ribosomal protein S2
RT-qPCR: Reverse transcription quantitative real-time polymerase chain reaction
S: Serine
SA: Salicylic acid
SnRK: SNF1-related protein kinase
SOBIR1: SUPPRESSOR OF BIR1–1
SRF: STRUBBELIG-RECEPTOR FAMILY
SSP: SHORT SUSPENSOR
STRE: Stress response element
TPR: Tetratricopeptide repeat
WAKL: WALL-ASSOCIATED RECEPTOR KINASE-LIKE
WGD: Whole genome duplication
WT: Wild-type
Y: Tyrosine
ZEP: Zeaxanthin epoxidase
