# Supplementary material for: Genome-wide identification and expression analysis reveals spinach brassinosteroid-signaling kinase (BSK) gene family functions in temperature stress response
Source: BMC Genomics. 2022 Jun 20;23:453. doi: 10.1186/s12864-022-08684-5 (PMC9208177; doi:10.1186/s12864-022-08684-5)
Supplement: Supplementary file 3 — Additional file 3. [file 12864_2022_8684_MOESM3_ESM.docx]

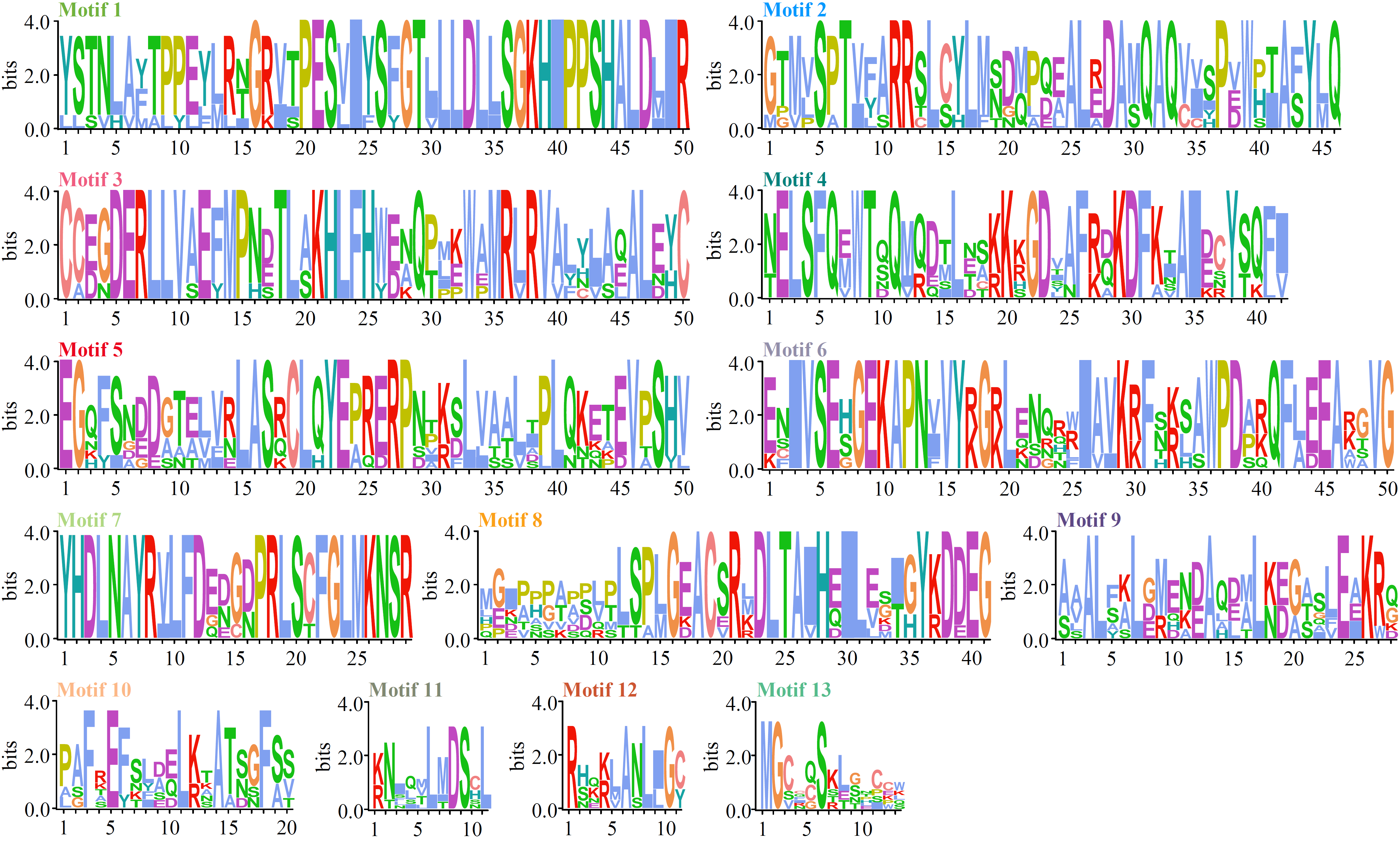


**Fig. S1** Sequence logos of thirteen motifs in SoBSKs. The height of each amino acid represented the relative frequency of the amino acid at that position.
