# Supplementary material for: Genome-wide identification and expression analysis reveals spinach brassinosteroid-signaling kinase (BSK) gene family functions in temperature stress response
Source: BMC Genomics. 2022 Jun 20;23:453. doi: 10.1186/s12864-022-08684-5 (PMC9208177; doi:10.1186/s12864-022-08684-5)
Supplement: Supplementary file 7 — Additional file 7. [file 12864_2022_8684_MOESM7_ESM.docx]

**Table S6.** List of primers used in cloning and RT-qPCR analysis for *SoBSKs*.

| **Gene name** | **Primer name** | **Primer sequence (5’-3’)** |
| --- | --- | --- |
| *Primers for cloning* | | |
| *SoBSK1* | SoBSK1-F | ATGGGGTGTTTTCAATCAACATTGG |
|  | SoBSK1-R | TCAGCTTCCTTTCATACTGCGT |
| *SoBSK2* | SoBSK2-F | ATGGGCTGCATACAGTCCAA |
|  | SoBSK2-R | TCAGCCTCGCCAGCTACTTT |
| *SoBSK3* | SoBSK3-F | ATGGGAGTTCGTTGCTCAAA |
|  | SoBSK3-R | TTAGTTTTTCTTCCATTTGGCCTCC |
| *SoBSK4* | SoBSK4-F | ATGGGATCCCAAGGATCCAAG |
|  | SoBSK4-R | TTATTTTGCATTGGTAGCATCCCT |
| *SoBSK5* | SoBSK5-F | ATGGGCTGTAACTGCTCACG |
|  | SoBSK5-R | CTATCCGGTACTACCTCGTTTAAT |
| *SoBSK6* | SoBSK6-F | ATGATTAACTTGCTGTCTGT |
|  | SoBSK6-R | CTAACTTCTTCCGCCTTTCTGC |
| *SoBSK7* | SoBSK7-F | ATGGGGTGCTGTCAATCCTC |
|  | SoBSK7-R | CTAACTTCTTCCGCCTTTCTGC |
| *Primers for RT-qPCR* | | |
| *SoBSK1* | q-SoBSK1-F | TTTGTTCTCAGACCAGCCCG |
|  | q-SoBSK1-R | GGCTGCATCTTTGTGCATGT |
| *SoBSK2* | q-SoBSK2-F | TCCGACAAGCAAATCTCAGCA |
|  | q-SoBSK2-R | ACATTAGGTGCTTTCTCCCCA |
| *SoBSK3* | q-SoBSK3-F | GGTGAGTCACAGCCGAGTTT |
|  | q-SoBSK3-R | GGCCATCATTGAGCTTCCCA |
| *SoBSK4* | q-SoBSK4-F | TCGTCCGTGCCTTTATCTGC |
|  | q-SoBSK4-R | CGCCACTCCTTCGTCATCTT |
| *SoBSK5* | q-SoBSK5-F | GCTGTAACTGCTCACGCCTA |
|  | q-SoBSK5-R | TGAATTCGCGAAATGCTGGC |
| *SoBSK6* | q-SoBSK6-F | GCTGTCTGTACATGTGATGGC |
|  | q-SoBSK6-R | TCAAGAGCATGGCTTGGAGG |
| *SoBSK7* | q-SoBSK7-F | GGGGTGCTGTCAATCCTCAG |
|  | q-SoBSK7-R | AAAGCAGGAACTCCACCAGC |
| *SoARF* | q-SoARF-F | CCGATAAGCTTGGCCTCCAT |
|  | q-SoARF-R | AGCCTTGCTAGCGATGTTGT |
